# Retraction: Phytoremediation of nickel by quinoa: Morphological and physiological response

**DOI:** 10.1371/journal.pone.0273531

**Published:** 2022-08-31

**Authors:** The PLOS ONE Editors

The *PLOS ONE* Editors retract this article [1] because it was identified as one of a series of submissions for which we have concerns about authorship, competing interests, and peer review. We regret that the issues were not addressed prior to the article’s publication.

QA agreed with retraction. MH, SI, MBH, MSS, NZ, AR, MUI, TJ, HMA, and MHS did not agree with the retraction. JI and MK either did not respond directly or could not be reached.
